# Supplementary material for: Sexual Orientation Discrimination and Exclusive, Dual, and Polytobacco Use among Sexual Minority Adults in the United States
Source: Int J Environ Res Public Health. 2022 May 23;19(10):6305. doi: 10.3390/ijerph19106305 (PMC9142070; doi:10.3390/ijerph19106305)
Supplement: Supplementary file 1 [file ijerph-19-06305-s001.zip › ijerph-1691150-supplementary.pdf]

Table S1. Weighted Proportions of Sexual Minority Status Indicators by Sex Among the Full and Analytic Samples of Sexual Minority Adults Asked About Sexual Orientation Discrimination

	Full Sample			Analytic Sample		
		By Sex			By Sex	
	Overall (*n* = 36,309)	Female (*n* = 20,447)	Male (*n* = 15,862)	Overall (*n* = 3453)	Female (*n* = 2215)	Male (*n* = 1238)
Sexual attraction, *n* (%)						
Only attracted to females	15054 (45.7)	530 (2.5)	14524 (92.3)	556 (15.9)	348 (15.3)	208 (17.0)
Mostly attracted to females	544 (1.5)	115 (0.5)	429 (2.6)	531 (17.2)	114 (4.8)	417 (36.4)
Equally attracted to females and males	582 (1.4)	444 (2.0)	138 (0.7)	571 (15.3)	438 (19.1)	133 (9.6)
Mostly attracted to males	1074 (2.7)	933 (4.4)	141 (0.8)	1040 (29.8)	903 (42.3)	137 (10.6)
Only attracted to males	18720 (47.8)	18228 (89.6)	492 (2.8)	636 (18.4)	339 (15.3)	297 (23.1)
Unknown	335 (0.9)	197 (0.9)	138 (0.8)	119 (3.3)	73 (3.3)	46 (3.4)
Sexual behavior, *n* (%)						
Only males	18871 (48.0)	18353 (89.9)	518 (2.8)	1221 (35.0)	897 (42.2)	324 (24.0)
Only females	14710 (44.7)	332 (1.6)	14378 (91.1)	576 (18.1)	165 (6.9)	411 (35.3)
Both males and females	1455 (3.6)	1040 (4.6)	415 (2.5)	1424 (40.2)	1022 (44.6)	402 (33.5)
Never had sex	908 (2.8)	503 (2.8)	405 (2.8)	104 (3.1)	51 (2.6)	53 (4.0)
Unknown	365 (0.9)	219 (1.1)	146 (0.8)	128 (3.5)	80 (3.7)	48 (3.2)
Sexual orientation, *n* (%)						
Heterosexual	34644 (95.9)	19454 (95.6)	15190 (96.3)	2041 (60.8)	1364 (63.4)	677 (56.7)
Lesbian or gay	586 (1.5)	265 (1.2)	321 (1.8)	577 (16.4)	261 (11.3)	316 (24.3)
Bisexual	566 (1.3)	422 (1.8)	144 (0.8)	553 (15.0)	415 (17.8)	138 (10.7)
Not sure	199 (0.5)	130 (0.6)	69 (0.4)	189 (5.3)	124 (5.3)	65 (5.3)
Unknown	314 (0.8)	176 (0.8)	138 (0.8)	93 (2.5)	51 (2.2)	42 (2.9)

Table S2. Weighted Prevalence of Past-Year Exclusive, Dual, and Polytobacco Product Use Among Sexual Minority Adults (n = 3453)

	n (%)
16-category variable	
Never/former	2217 (64.8)
Exclusive cigarette	886 (24.3)
Exclusive ENDS	10 (0.3)
Exclusive other combustible (OC)	47 (1.4)
Exclusive smokeless tobacco (SLT)	22 (0.7)
Dual cigarette + ENDS	150 (4.8)
Dual cigarette + OC	63 (1.8)
Dual cigarette + SLT	15 (0.5)
Dual ENDS + OC	3 (0.1)
Dual ENDS + SLT	0 (0.0)
Dual OC + SLT	2 (0.1)
Poly cigarette + ENDS + OC	21 (0.5)
Poly cigarette + ENDS + SLT	8 (0.3)
Poly cigarette + OC + SLT	6 (0.3)
Poly ENDS + OC + SLT	0 (0.0)
Poly cigarette + ENDS + OC + SLT	3 (0.1)
4-category variable	
Never/former	2217 (64.8)
Exclusive use	965 (26.7)
Dual use	233 (7.3)
Polyuse	38 (1.2)

ENDS: Electronic nicotine delivery systems

OC: Other combustibles (cigars or traditional pipe)

SLT: Smokeless tobacco

Table S3. Weighted Means of Prior-to-Past-Year Experiences of Sexual Orientation Discrimination Overall and by Past-Year Exclusive, Dual, and Polytobacco Use With Pairwise Comparisons Between Tobacco Use Groups (n = 3453)

	Past-Year Tobacco Product Use							
	Never/Former Use (n = 2,217)	Exclusive Use (n = 965)	Dual Use (n = 233)	Polyuse (n = 38)	P [a]	P [b]	P [c]	P [d]
Sexual orientation discrimination items, mean (95% CI) [e]								
Obtaining health care or insurance	0.10 (0.08, 0.12)	0.09 (0.06, 0.13)	0.16 (0.06, 0.26)	0.47 (0.06, 0.89)	0.09	0.24	0.07	0.13
Receiving health care	0.10 (0.08, 0.12)	0.10 (0.07, 0.14)	0.13 (0.05, 0.22)	0.36 (-0.06, 0.78)	0.18	0.56	0.22	0.28
While in public places	0.23 (0.20, 0.27)	0.27 (0.20, 0.33)	0.38 (0.26, 0.50)	0.52 (0.06, 0.97)	**0.013**	0.08	0.26	0.56
While in other situations	0.13 (0.10, 0.16)	0.14 (0.10, 0.18)	0.21 (0.10, 0.32)	0.20 (-0.03, 0.42)	0.16	0.25	0.61	0.91
Called names	0.26 (0.22, 0.30)	0.30 (0.23, 0.38)	0.44 (0.32, 0.55)	0.46 (0.13, 0.79)	**0.003**	0.06	0.34	0.91
Bullied, assaulted, or threatened	0.17 (0.13, 0.20)	0.18 (0.12, 0.24)	0.30 (0.19, 0.42)	0.30 (0.06, 0.55)	0.06	**0.049**	0.29	0.98
Sexual orientation discrimination scale [e]	0.99 (0.83, 1.14)	1.09 (0.82, 1.36)	1.62 (1.08, 2.16)	2.31 (0.83, 3.78)	**0.014**	0.08	0.09	0.36

[a] ANOVA test comparing means of each discrimination item across tobacco product use groups

[b] Pairwise t-test comparing means of each discrimination item between exclusive use and dual use

[c] Pairwise t-test comparing means of each discrimination item between exclusive use and polyuse

[d] Pairwise t-test comparing means of each discrimination item between dual use and polyuse

[e] Each sexual orientation discrimination item ranges from 0-4; the sexual orientation discrimination scale ranges from 0-24

Bold p-values are statistically significant ($p < 0.05$)

Table S4. Multivariable Multinomial Logistic Regression Models of Associations Between Past-Year Experiences of Sexual Orientation Discrimination and Past-30-Day Exclusive, Dual, and Polytobacco Use (n = 3453)

	Past-30-Day Tobacco Product Use [a]			
	Exclusive Use		Dual/Polyuse	
	AOR [b]	95% CI	AOR [b]	95% CI
Sexual orientation discrimination items				
Obtaining health care or insurance	1.05	0.82, 1.33	1.14	0.78, 1.69
Receiving health care	0.92	0.72, 1.18	1.11	0.75, 1.64
While in public places	1.13	0.98, 1.31	1.21	0.93, 1.56
While in other situations	1.08	0.88, 1.32	1.28	0.89, 1.83
Called names	1.11	0.97, 1.28	1.31	1.00, 1.72
Bullied, assaulted, or threatened	**1.30**	**1.03, 1.63**	**1.65**	**1.18, 2.31**
Sexual orientation discrimination scale	1.03	0.99, 1.07	1.07	1.00, 1.15

[a] The outcome referent group: never/former use

[b] Adjusted odds ratios (OR) and 95% confidence intervals (CI) adjusted for mean-centered age, quadratic age, sex, race/ethnicity, highest educational attainment, annual household income, urbanicity, and geographic region

Bold AORs and 95% CIs are statistically significant ($p < 0.05$)

Table S5. Two-Way Interaction Terms Between Prior-to-Past-Year Experiences of Sexual Orientation Discrimination and Sex (*n* = 3453)

Two-Way Interaction Terms [a]	P [b]
Sexual orientation discrimination items	
Obtaining health care or insurance*sex	0.16
Receiving health care*sex	0.24
While in public places*sex	0.22
While in other situations*sex	0.89
Called names*sex	0.63
Bullied, assaulted, or threatened*sex	0.69
Sexual orientation discrimination scale*sex	0.52

[a] Each discrimination measure is continuous; the referent group for sex is male (vs. female)

[b] Wald test p-value for interaction

Two-way interactions were tested in adjusted associations between prior-to-past-year experiences of sexual orientation discrimination and past-year exclusive, dual, and polytobacco use

Table S6. Multivariable Multinomial Logistic Regression Models of Associations Between Prior-to-Past-Year Experiences of Sexual Orientation Discrimination and Past-Year Exclusive, Dual, and Polytobacco Use Stratified by Sexual Minority (SM) Status and Sex (n = 3106)

	Past-Year Tobacco Product Use [a]			
	Exclusive Use		Dual/Polyuse	
	AOR [b]	95% CI	AOR [b]	95% CI
Prior-to-past-year sexual orientation discrimination scale				
By SM status and sex [c]				
Heterosexual women with same-sex attraction and/or behavior (n = 1,314)	0.97	0.88, 1.07	1.12	0.96, 1.32
Heterosexual men with same-sex attraction and/or behavior (n = 662)	1.09	0.95, 1.25	**1.23**	**1.05, 1.44**
SM-identified women (n = 676)	1.01	0.94, 1.08	1.02	0.94, 1.10
SM-identified men (n = 454)	0.94	0.88, 1.01	1.08	0.98, 1.19

[a] The outcome referent group: never/former use

[b] Adjusted odds ratios (OR) and 95% confidence intervals (CI) adjusted for mean-centered age, quadratic age, sex, race/ethnicity, highest educational attainment, annual household income, urbanicity, and geographic region

[c] SM-identified includes identifying as gay or bisexual for men and lesbian or bisexual for women respondents

Bold AORs and 95% CIs are statistically significant ($p < 0.05$)
